# Editor's Letter-Box

**Published:** 1902-01-11

**Authors:** 


### EDITOR'S LETTER-BOX.

[Our Correspondents are reminded that prolixity is a great bar to publication, and that brevity of style and conciseness of statement greatly facilitate early insertion.]

*To the Editor of* THE HOSPITAL.

MISS MARY GARDNER, lady superintendent of the Birmingham and Midland Counties Sanatorium, near Bromsgrove, writes:—

DEAR SIR,—Your suggestion that the opinion of leading medical women should be sought on the question of the nature and extent of work undertaken by junior medical women seems eminently just, and I hope some movement will be made to obtain this. But the opinion of Mrs. Garrett Anderson may be inferred beforehand, from the fact that her daughter lately held the post of junior house-surgeon in one of the principal metropolitan infirmaries.

